# Cryptotanshinone activates AMPK-TSC2 axis leading to inhibition of mTORC1 signaling in cancer cells

**DOI:** 10.1186/s12885-016-3038-y

**Published:** 2017-01-07

**Authors:** Wenxing Chen, Yanhong Pan, Siliang Wang, Yuping Liu, Guangying Chen, Liang Zhou, Wenting Ni, Aiyun Wang, Yin Lu

**Affiliations:** 1 School of Pharmacy, Nanjing University of Chinese Medicine, NO.138, Xianlin Avenue, Nanjing, Jiangsu Province 210023 China; 2 Jiangsu Key Laboratory for Pharmacology and Safety Evaluation of Chinese Materia Medica, Nanjing University of Chinese Medicine, Nanjing, Jiangsu Province 210023 China; 3 College of Chemistry and Chemical Engineering, Hainan Normal University, Haikou, 571158 China

**Keywords:** Cryptotanshinone, AMPK, TSC2, mTORC1

## Abstract

**Background:**

Cryptotanshinone (CPT), a fat-soluble phenanthraquinone from *Salvia miltiorrhiza* Bunge, has been demonstrated to inhibit phosphorylation of p70 S6 kinase 1 (S6K1) and eukaryotic initiation factor 4E binding protein 1 (4E-BP1), a couple of direct downstream effectors of the mammalian target of rapamycin complex 1 (mTORC1), resulting in cancer cell arrested in G0 phase and subsequent inhibition of proliferation. However, its concrete molecular mechanism about how CPT inhibits mTORC1 signaling pathway is unclear.

**Methods:**

one solution was used to check cell viability and western blotting for determining expression of the indicated proteins. Molecular docking was performed to assess the binding of CPT with mTOR. The co-immunoprecipitation assay was to analyze whether CPT could disrupt the mTORC1 and TSC1/TSC2 complex. Recombinant adenoviral dominant-negative AMPKα was used to downregulate expression of AMPKα and lentiviral AMPK and TSC2 to silence the AMPK and TSC2 in Rh30 cells.

**Results:**

Primarily, Rh30 cells expressing rapamycin-resistant mutant mTOR are also sensitive to CPT, while the molecular docking result for CPT binding to mTOR is negative, suggesting that CPT inhibition of mTORC1 is different from rapamycin. Then the related proteins of PTEN-PI3K pathway was proved not to be affected, but the phosphorylation of adenosine monophosphate-activated protein kinase (AMPK) was activated by a concentration- and time- dependent manner, meaning that it may be associated with AMPK. Further results indicated that compound C, inhibitor of AMPK, could clearly reversed CPT inhibitory effect on Rh30 cells, and dominant-negative AMPK in cancer cells conferred resistance to CPT inhibition of 4E-BP1 and phosphorylation of S6K1, as well as sh-AMPK. Furthermore, compared with AMPK-positive MEF cells, AMPK-negative MEF cells are less sensitive to CPT by the findings that 4E-BP1 and phosphorylation of S6K1 express comparatively more. Additionally, phosphorylation of tuberous sclerosis complex 2 (TSC2) was activated under the treatment of CPT, and down-expression of TSC2 by shRNA slightly recovered expression of 4E-BP1 and phosphorylation of S6K1, while co-immunoprecipitation of TSC2 did not alter expression of TSC1 by CPT.

**Conclusion:**

CPT inhibiting mTORC1 pathway was mostly due to activation of AMPK-TSC2 axis rather than specific binding to mTORC1. CPT is a potent anticancer agent targeting AMPK.

## Background

The mTOR, a 289-KD serine/threonine protein kinase, plays a core role in multiple signaling pathways involving the mediation of cell growth, proliferation, apoptosis and autophagy, being considered a hotspot target for cancer therapy [1, 2]. mTOR has a pair of multiprotein complex forms including mTOR complex 1 and 2 (mTORC1 and mTORC2) [3, 4]. mTORC1 activation results in phosphorylation of 4EBP1 and S6K1, regulating cell proliferation [3, 5], while mTORC2 phosphorylates Akt at Ser473 and probably promotes Akt phosphorylation by regulating integrin related kinase [3, 6]. mTORC2 also controls actin cytoskeleton and cell migration via regulating phosphorylation of PKC [3, 6].

Activation of the mTOR signaling is firstly initiated by a variety of cellular signals comprising mitogenic growth factors, hormones, nutrients, cellular energy, stress conditions and so on, then initiative signals are transmitted by many important node molecules via multiple pathways to regulate mTOR in final [2]. A key pathway activating mTOR is the PI3K/Akt, which critically mediates cell survival and proliferation and is started by mitogenic stimuli from the binding of growth factors with receptors in the cell membrane [7–9]. Akt can directly phosphorylate mTOR or indirectly act on mTOR through complex of TSC1/TSC2 [2, 9]. Then the functional complex TSC1/TSC2 exerts an inhibition on mTORC1 [2, 9].

Apart from the PI3K/AKT, AMPK, another important signaling pathway can also modulate mTORC1 [10]. AMPK, a key energy-sensitive kinase, sensitively responds to the cellular AMP/ATP ratio [11]. AMPK is phosphorylated following the increase of the ratio and also activated by the upstream kinase LKB1, a human tumor suppressor with high mutation in Peutz-Jeghers syndrome [11]. Activation of AMPK in turn phosphorylates TSC2 obviously, which subsequently suppresses mTORC1 activity [10, 11].

Cryptotanshinone (CPT) is one of the four major fat-soluble tanshinones abstracted from Salvia miltiorrhiza Bunge widely used in traditional formula for treating a variety of diseases [12, 13]. Up to the present, cumulative data has demonstrated that CPT exhibits significant inhibitory effect in many cancer cells and should be a potential anticancer agent [13]. Previously, we have demonstrated that CPT could down-regulate expression of cyclin D1 and Rb protein and lead to cancer cell arrested in G0 phase [14], which is mainly mediated by inhibition of mTORC1 because the best-characterized effectors downstream of mTORC1, S6K1 and 4EBP1 were dephosphorylated. However, its concrete molecular mechanism about how CPT inhibits mTORC1 signaling is unclear.

Here, we demonstrated by the molecular docking data that CPT unlike rapamycin has weak direct affinity with FKBP12, the structure domain of mTOR. Concurrently, CPT indicates no inhibition on PI3K and PTEN. Further results conclusively confirmed that activation of AMPK-TSC2 axis is dependent for CPT inhibition of mTORC1.

## Methods

### Materials

Cryptotanshinone [1,2,6,7,8,9-hexahydro-1,6,6-trimethyl-phenanthro(1,2-b) furan-10,11-dione] was supplied by Xi’an Hao-Xuan Bio-Tech Co., Ltd. It was dissolved in anhydrous ethanol to form the stock solutions (20 mmol/L) stored at -20 °C. RPMI 1640 and Dulbecco’s Modified Eagle Medium (DMEM) were purchased from Mediatech (Herndon, VA, USA). Fetal bovine serum (FBS) was from Hyclone (Logan, UT, USA), and 0.05% Trypsin-EDTA from Invitrogen (Grand Island, NY, USA). Type I insulin-like growth factor (IGF-1) (PeproTech, Rocky Hill, NJ, USA) was rehydrated in 0.1 M acetic acid to prepare a stock solution (10 μg/ml) for equipartition and storage at -80 °C. Enhanced chemiluminescence solution was from Perkin Elmer Life Science (Boston, MA, USA). The antibodies used includes: 4E-BP1 (Zymed, South San Francisco, CA, USA), phospho-S6K1 (Thr389), S6K1, cyclin D1, Rb, AMPK, phospho-AMPKα(Thr172), ACC, phospho-ACC(Ser79), PDK1, phospho-PDK1(Ser241), PI3K(p110), PI3K(p85), phospho-PI3K(p85), PTEN, phospho-PTEN(Ser380), TSC1, TSC2, phospho-TSC2(Thr1462), (Santa Cruz Biotechnology, Santa Cruz, CA, USA), phospho-mTOR(Ser2448), mTOR, p-4E-BP1(T37/46), raptor, phospho-raptor(Ser792), rictor, mLST8 (Cell Signaling, Beverly, MA, USA), AU1, HA (Bethyl Laboratories, Montgomery, TX, USA), Flag, β-tubulin (Sigma, St. Louis, MO, USA).

### Cell lines and cultures

Human rhabdomyosarcoma (Rh30), human prostate carcinoma (DU-145), human breast cancer (MCF-7), MEF (AMPK^+/+^) and MEF (AMPK^-/-^) cells (mouse embryonic fibroblast cells) were generously provided by Dr. Shile Huang (Louisiana State University Health Science Center, Shreveport, LA, USA). Rh30, DU-145 and MCF-7 cells were cultured in antibiotic-free RPMI 1640 medium plus with 10% FBS, while MEF cells in antibiotic-free DMEM containing 10% FBS. All cells were maintained in a humid incubator (37 °C, 5% CO_2_). For serum-free experiments, monolayer cells were washed with fresh phosphate-buffered saline (PBS) and cultured in the serum-free DMEM.

### Cell proliferation assay

Cells dispersed evenly in the medium were seeded in a 96-well plate at a density of 1 × 10^4^ cells/well (6 replicates) and grown overnight at 37 °C in a humidified incubator with 5% CO_2_. After CPT treatment for 48 h, 20 μL of one solution reagent was added into each well and the 96-well plate was continuously incubated for 4 h. Cell proliferation was determined by reading the optical density (OD) at 490 nm using a Wallac 1420 Multilabel Counter (Perkin-Elmer Life Sciences, Wellesley, MA, USA).

### Molecular docking

Searching and downloading the protein crystal structure of mTOR (2NPU) in the database of protein data bank (PDB) (http://www.rcsb.org/pdb/home/home.do). And downloading the 3D structure of rapamycin (CID:5284616), an inhibitor of mTOR and a positive control, and the docking molecule, cryptotanshinone (CID:496348) in the database of pubmed compound (http://www.ncbi.nlm.nih.gov/pccompound). Then the dehydration, hydrogenation and dehydrogenation for the protein and the molecules were executed using chimera software. In Autodock4.2 software, the semi-empirical potential function as the energy scoring function was used to score the small molecule conformation and location followed with Lamarck (LGA) genetic algorithm combined with local energy search. The size of receptor lattice box for docking is 2.25 nm × 2.25 nm × 2.25 nm(60 point × 60 point × 60 point), the lattice interval is 0.375 nm. The center of lattice box is located in the center of active site of mTOR protein. In the docking results, Similarity and free Binding energy for the dock site between cryptotanshinone and rapamycin were compared and analyzed.

### Western blot analysis

Western blotting was performed as described previously [14]. In brief, cells were washed with cold PBS, then lysed in cold RIPA buffer. Lysates were sonicated for 10 s and centrifuged at 14,000 rpm for 10 min at 4 °C. After protein concentration was quantitated by bicinchoninic acid assay, equivalent amounts of protein mixed with loading buffer were loaded and separated on 7.5%-12% SDS-polyacrylamide gel. The separated protein was transferred to a polyvinylidene difluoride (PVDF) membrane which was then incubated with PBS containing 0.05% Tween-20 and 5% nonfat dry milk for blocking non-specific binding. Finally, the PVDF membrane was successively incubated with primary antibodies and appropriate secondary antibodies conjugated to horseradish peroxidase. Immunoreactive bands were visualized by enhanced chemiluminescence detection reagent.

### Co-immunoprecipitation

The immunoprecipitation assay was done as described previously [15]. Briefly, cells were grown in 100-mm dishes with appropriate 10% FBS medium for 2 d, and then the medium was replaced with serum-free DMEM for 24 h starvation. Serum-starved cells were exposed to CPT (2.5 μmol/L - 20 μmol/L) for 2 h with subsequent stimulation with 10 ng/mL IGF-I for 1 h. Then cells were washed once in ice-cold 1x PBS and lysed in different buffer. For the mTOR co-immunoprecipitation, cells were lysed in cold 1x Chaps buffer. 500 μL of cell lysates mixed with 1 μg of goat anti-mTOR antibody and 30 μL of protein A/G plus agarose were incubated overnight at 4 °C. After centrifugation, immunoprecipitates were washed four times with 1x Chaps buffer. Final samples were subjected to SDS-PAGE as described above. For the TSC1/2 co-immunoprecipitation, cells were lysed in cold NP40 lysis buffer. 500 μL of cell lysates mixed with 1 μg of rabbit anti-TSC2 antibody and 30 μL of protein A/G plus agarose were incubated overnight at 4 °C. After centrifugation, immunoprecipitates were washed four times with NP40 lysis buffer. Samples were subjected to western blotting.

### Recombinant adenovirus infects cells

Recombinant adenovirus encoding HA-tagged dominant negative AMPKα (Ad-dn-AMPKα) was a gift from Dr. Shile Huang (Louisiana State University Health Science Center, Shreveport, LA, USA). Initially, Rh30 cells were cultured in the growth medium, and the recombinant adenovirus was added for 24-h infection at 5 of multiplicity of infection (MOI = 5). Cells infected with Ad-GFP adenovirus alone served as a control. Expression of HA-tagged dn-AMPKα was confirmed by western blotting for HA band. The recombinant adenoviruses expressing FLAG-tagged wild-type mTOR, FLAG-tagged rapamycin-resistant (mTOR-rr) (mTORrr contains the entire mTOR gene with the site mutation (S2035I) that has intact mTOR kinase activity, but prevents rapamycin binding to FKBP12 and is rapamycin-insensitive [16, 17]) and the control vector expressing green fluorescence protein (GFP) alone (Ad-GFP) were from Dr. Shile Huang. All recombinant adenoviral constructs were amplified, titrated and used as described [16].

### Lentiviral shRNA assay

Lentiviral shRNAs to AMPKα, TSC2 and GFP were described previously [18]. For producing lentiviral shRNAs, above individual construct together with pMD2.G and psPAX2 (Addgene, Cambridge, MA) was jointly transfected into 293TD cells using Lipfectamine™ 2000 reagent. And the lentivirus-containing medium was respectively collected in 36 h and 60 h of post-transfection. When used, approximate 70% confluence of monolayer cells were infected by above lentivirus-containing supernatant in the presence of 8 μg/ml polybrene for 12 h twice at an interval of 6 h. The addition of 2 mg/ml puromycin for 48 h was used to eliminate uninfected cells before use [18].

### Statistical analysis

Quantitative data were expressed as Mean ± SE, and analyzed by one-way analysis of variance (ANOVA) with post-hoc Dunnett’s *t*-test for multiple comparisons. The statistical significant difference was considered when *p* < 0.05.

## Results

### CPT inhibits mTORC1 in an unlike-rapamycin manner

**Fig. 1:**
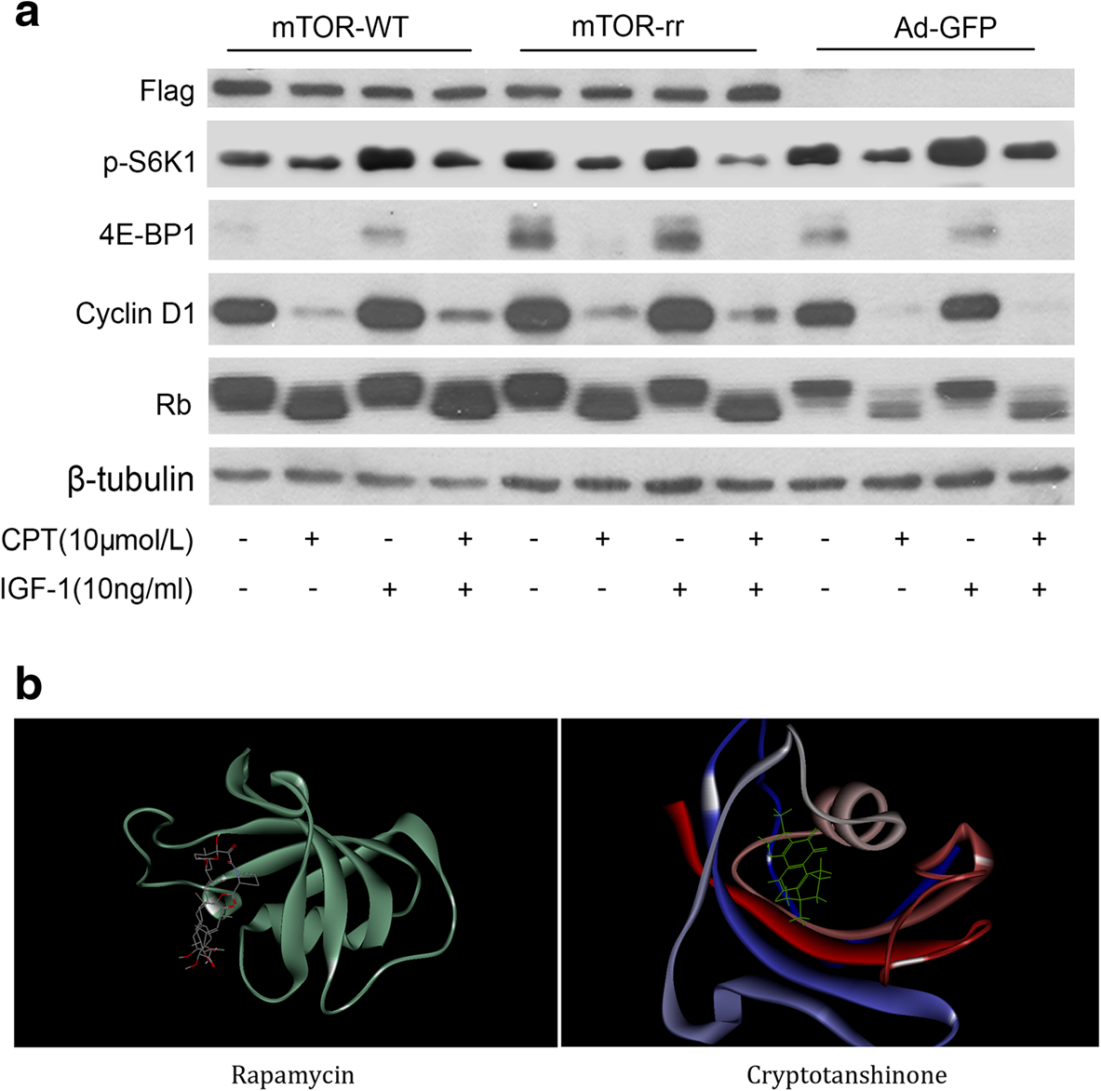
CPT inhibits mTOR pathway by an unlike-rapamycin way in cancer cells. **a** Serum-starved Rh30 cells respectively expressing rapamycin-resistant mTOR (mTORrr), mTOR-wt and ad-GFP, were pretreated with CPT (10 μmol/L) for 2 h, then stimulated with IGF-1 (10 ng/mL) for 1 h. The western blotting assay was used to check the following proteins expression: Flag, p-S6K1, 4E-BP1, Cycin D1, Rb and β-tubulin. **b** Molecular docking for CPT binding to mTOR

To understand whether CPT could directly binds to FKBP12, rapamycin binding domain of mTOR, Rh30 cells transfected with a rapamycin-resistant mutant mTOR (S2035I), mTORrr, that blocks FKBP12-rapamycin complex forming [17], were pretreated with CPT (10 μmol/L) and then induced by the presence or absence of 10 ng/ml IGF-1 which is one of key stimulators to initiate and activate the mTOR signaling [19]. The results shown in Fig. 1a indicated that expression of p-S6K1, 4E-BP1, cyclin D1 and Rb in Rh30 cells with mTOR-rr was also inhibited as usual, and no difference was noted between mTOR-WT and mTOR-rr Rh30 cells. Generally, if CPT could bind to FKBP12, obvious reversed effect on phosphorylation of S6K1 and 4E-BP1 would be detected, as well as expression of cyclin D1 and Rb in mTOR-rr transfected cells. Thus, the data basically declared that CPT inhibits mTORC1 independent of FKBP12. Furthermore, the molecular docking data using Autodock4.2 software also deduced the same results. As shown in Table 1, the CDOCKER energy (-69.294) of rapamycin binding mTOR is completely less than that of CPT (-0.443), as well as the CDOCKER interaction energy. And the Fig. 1b illustrated the binding of two compounds to mTOR. For compounds, the less energy, the more stable. Thus, above-mentioned results clued that CPT could not specifically bind to mTOR.

**Table 1:** Energy comparison of affinity to mTOR in two compounds

Compound	CDOCKER Energy^a^	CDOCKER Interaction Energy^a^
Rapamycin	−69.294	−80.192
Cryptotanshinone	−0.443	−29.761

^a^Less energy, more stable

### CPT does not affect PTEN-PI3K pathway

**Fig. 2:**
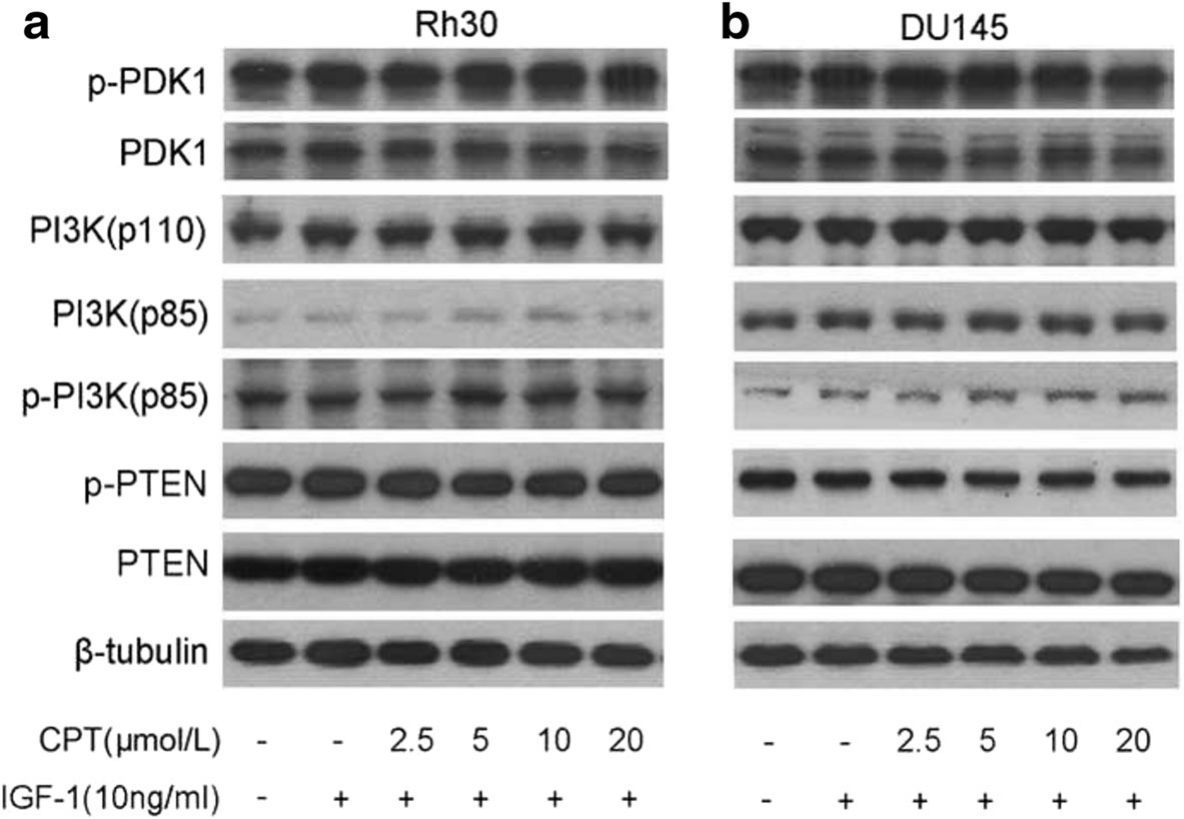
CPT does not decrease expression of p-PDK1, or p-PI3K p85, or increase expression of p-PTEN in cancer cells. Serum-starved Rh30 (**a**) or DU145 (**b**) cells were firstly treated with CPT (0-20 μmol/L) for 2 h, then stimulated with IGF-1 (10 ng/mL) for 1 h, followed by western blotting assay for the indicated proteins expression

PI3K, a bona fide upstream positive regulator of mTORC1, was routinely considered to mediate CPT inhibition of mTORC1 by deactivation. However, in the previous paper, we have found that CPT could up-regulate the expression of p-AKT (S473 & T308), leading to activation of AKT [14]. Here, the data in Fig. 2a indicated that PI3K-p110 and PI3K-p85 express constant in CPT-treated cells, as well as p-PI3K-p85. Thus, it is suggested that PI3K is not involved in CPT inhibition of mTORC1. Similarly, the PDK1, downstream of PI3K and directly activated by PI3K, also has no response to CPT by intact expression of total PDK1 and p-PDK1 (Fig. 2a). In an addition, PTEN, another most commonly mutated tumor suppressor in humans, negatively regulates PI3K and mTORC1 through catalyzing phosphatidylinositol-4,5-bisphosphate (PIP2) to phosphatidylinositol-3,4,5-trisphosphate (PIP3), and thus possibly involves in CPT inhibition of mTORC1. However, no distinct difference was found from the western blot results about PTEN and p-PTEN. Furthermore, the same results were observed in DU145 cells. Therefore, CPT inhibition of mTORC1 is not related with PTEN-PI3K pathway. And the mechanism about CPT activation of AKT should be further elucidated in the future work.

### CPT does not disrupt mTORC1 formation

**Fig. 3:**
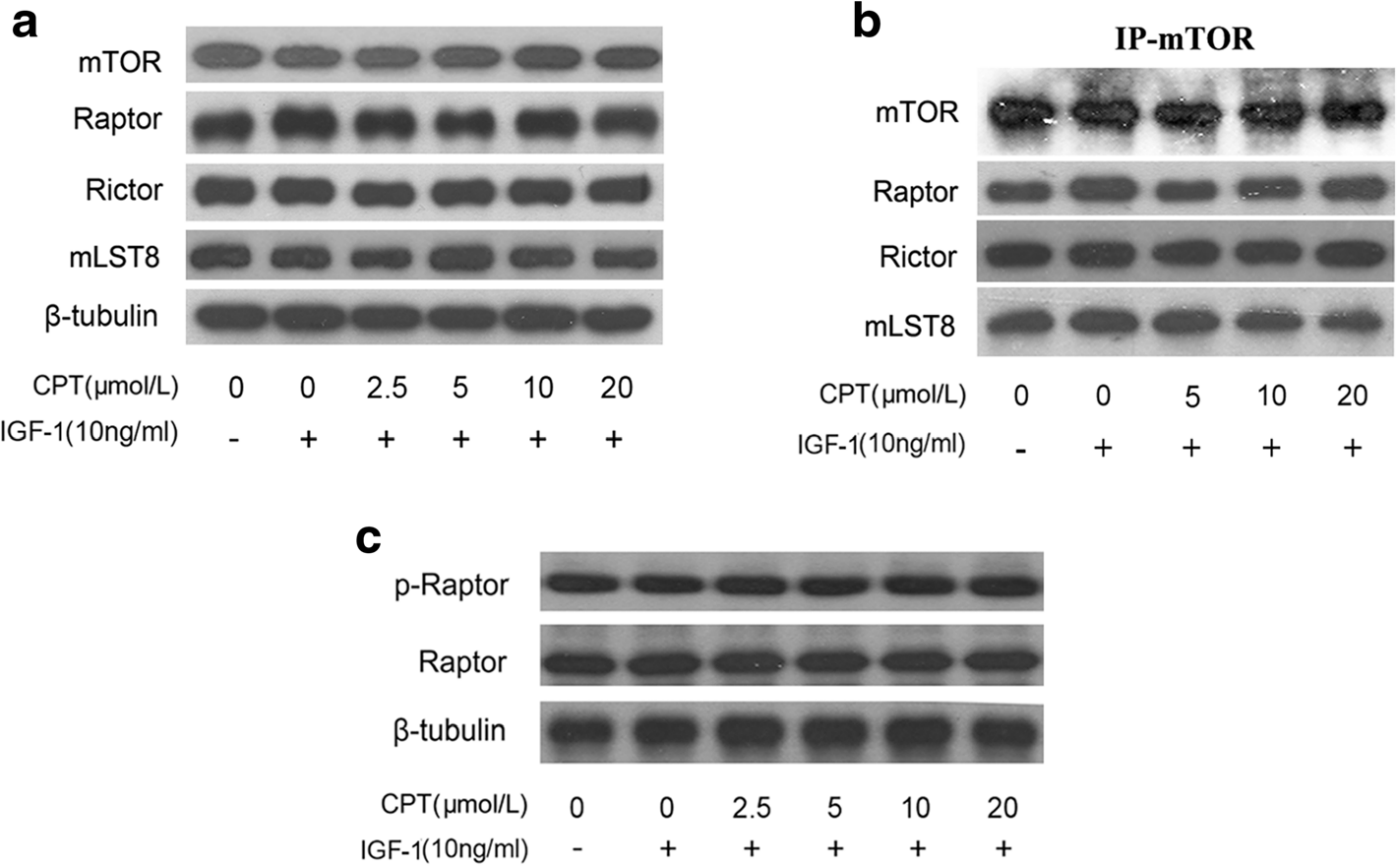
CPT does not affect mTORC1 formation. Serum-starved Rh30 cells were firstly exposed to CPT (0-20 μmol/L) for 2 h, then stimulated with IGF-1 (10 ng/mL) for 1 h, followed by western blotting assay for the indicated proteins expression (**a**, **c**), or by immunoprecipitation with antibody to mTOR plus protein A/G agarose, and immunoblotting with antibodies to mTOR, raptor, rictor and mLST8 (**b**)

The major binding proteins of mTORC1 include mTOR, mLST8 and raptor [5]. Rapamycin dissociates raptor from mTORC1 leading to inhibition of mTOR activity [5], and the natural compound, curcumin also was demonstrated to disrupt mTORC1 complex to exert its inhibition of mTOR [15]. To confirm whether CPT inhibits mTORC1 via dissociating mTOR, mLST8, raptor and rictor, mTOR was immunoprecipitated from the lysates of Rh30 cells treated with CPT and IGF-1, followed by immunoblotting for mTOR, raptor, rictor and mLST8, respectively. As shown in Fig. 3, neither cellular protein levels of raptor, rictor and mLST8 (Fig. 3a) nor expression of raptor, rictor and mLST8 when immunoprecipitates mTOR (Fig. 3b) in Rh30 cells were obviously changed by CPT, suggesting CPT does not disrupt the mTORC1 and mTORC2 complex. Moreover, raptor is also directly phosphorylated by AMPK to disrupt mTORC1 for controlling protein translation [20]. In Fig. 3c, CPT could not affect expression of p-raptor and total raptor, meaning that the direct phosphorylation of raptor was excluded for CPT inhibition of mTORC1 pathway.

### CPT activates AMPK pathway

**Fig. 4:**
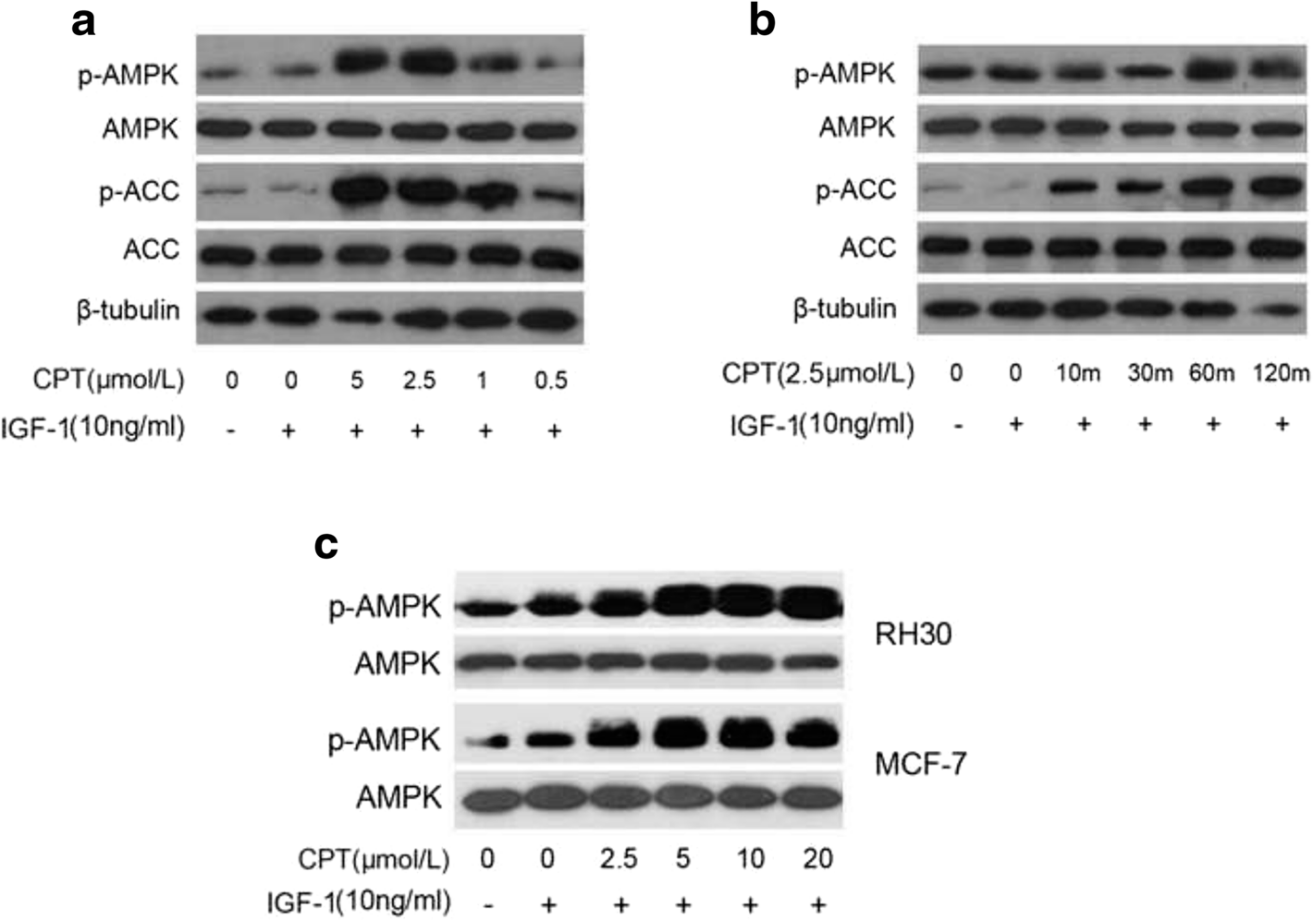
CPT activates AMPK in a concentration and time dependent manner in cancer cells. Serum-starved Rh30 cells were firstly exposed to CPT (0-5 μmol/L) for 2 h (**a**) or to CPT (2.5 μmol/L) for the indicated time (**b**), then stimulated with IGF-1 (10 ng/mL) for 1 h, followed by western blotting assay for proteins expression of p-AMPK, AMPK, p-ACC, ACC. **c** The same experiments were repeated in MCF-7 cells and Rh30 cells by CPT (0-20 μmol/L)

Since CPT has no affinity with FKBP12 and no effect on inhibiting the PTEN-PI3K pathway and disrupting the mTORC1, its inhibition of mTOR pathway then maybe due to intervention of upstream of mTOR, such as AMPK. So the expression of above protein kinase in cancer cells was measured using western blot assay. As shown in Fig. 4a, 1 μmol/L CPT could dominantly activate the phosphorylation of AMPK in Rh30 cells, as well as the phosphorylation of ACC, which is concentration-dependent. And Fig. 4b showed that the activation reaction occurred by a time-dependent manner and the phosphorylation of ACC increased significantly in ten minutes while p-AMPK in one hour after CPT treated cancer cells, and the effect would be continued for more than 2 h. Furthermore, similar results could be repeated in other cancer cells such as MCF-7, and in higher concentrations (Fig. 4c).

**Fig. 5:**
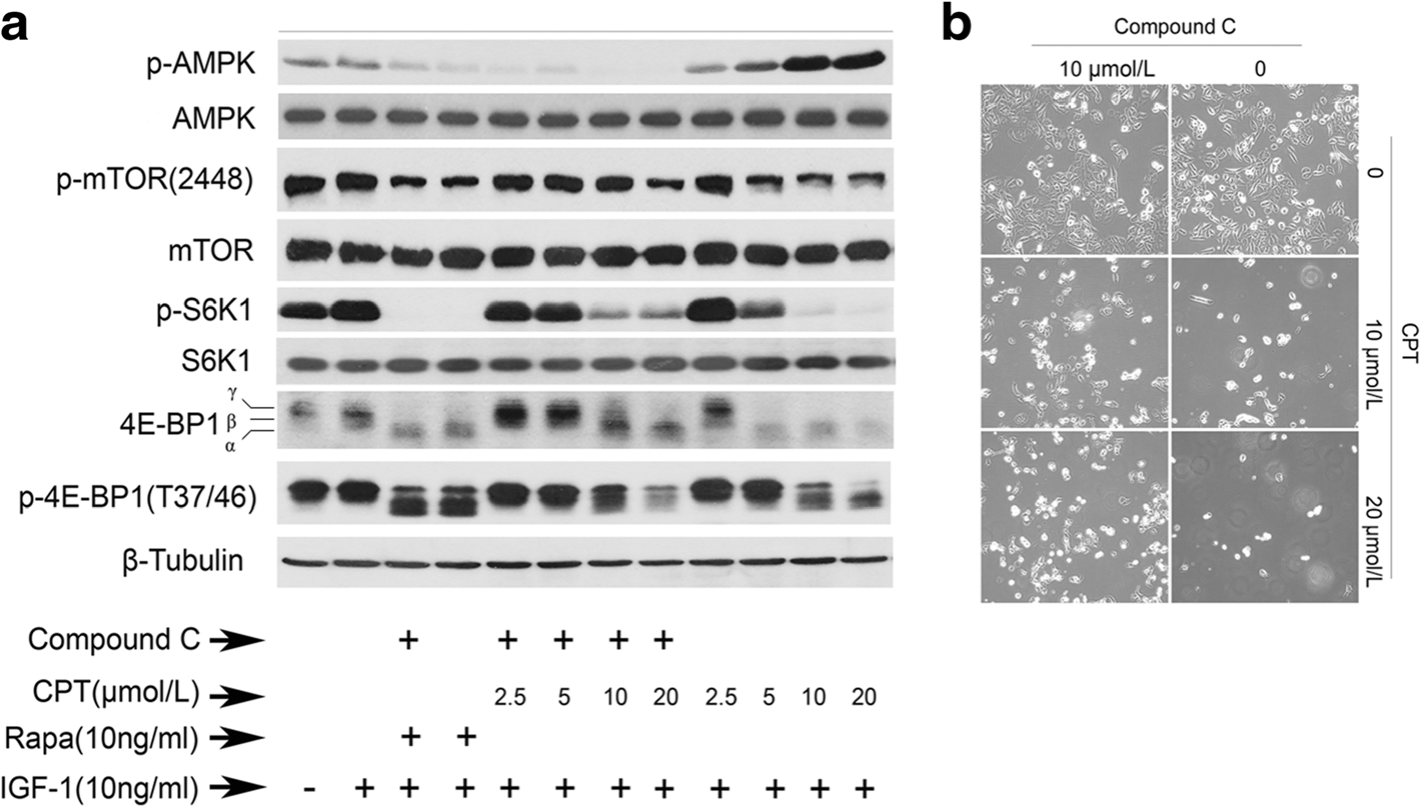
Inhibition of AMPK with Compound C attenuates CPT-induced inhibition of mTORC1 and cell growth. **a** Serum-starved Rh30 cells were firstly exposed to Compound C (10 μmol/L) for 0.5 h, and then treated with CPT (0-20 μmol/L) for 2 h, finally stimulated with IGF-1 (10 ng/mL) for 1 h, followed by western blotting assay for proteins expression. **b** Serum-starved Rh30 cells were firstly exposed to Compound C (10 μmol/L) for 0.5 h, then treated with CPT (0-20 μmol/L) for 48 h, followed by taking photograph using Image system

Compound C, inhibitor of AMPK, was added to further demonstrate CPT inhibiting mTORC1 was due to activation of AMPK. Based on Fig. 5a, high expression of p-AMPK induced by CPT was nearly undetected under the treatment of 10 μmol/L compound C, while expression of p-mTOR(2448) and p-S6K1 were both partially recovered, and the band shift of 4E-BP1 at 5 μmol/L CPT was completely inhibited by compound C and partially at 10 μmol/L CPT. However, similar results were not induced by rapamycin. The number of survival cells by CPT plus compound C is significantly more than by CPT alone in Fig. 5b, indicating that CPT inhibitory effect on Rh30 cells was attenuated by compound C. Thus, CPT inhibition of mTORC1 pathway was related to activation of AMPK.

### Both dominant-negative AMPKα and sh-AMPKα attenuates CPT inhibition on cancer cells

**Fig. 6:**
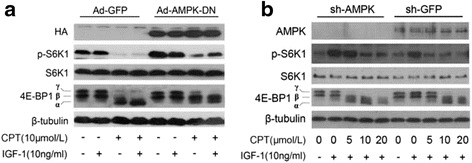
Expression of dominant negative AMPKα or down-regulation of AMPKα attenuates CPT-induced inhibition of mTORC1 signaling. Serum-starved Rh30 cells respectively expressing ad-GFP, and ad-AMPK-DN (**a**), or sh-AMPK and sh-GFP (**b**), were firstly exposed to CPT (10 μmol/L) for 2 h, then stimulated with IGF-1 (10 ng/mL) for 1 h, followed by western blotting assay for proteins expression of HA, p-mTOR(2448), mTOR, p-S6K1, S6K1, 4E-BP1, p-4E-BP1(T70). And β-tubulin was a control

To attain the further evidence on CPT activation of AMPK, Rh30 cells were infected with Ad-AMPKα-dn and lentiviral sh-AMPKα to downregulate the expression of AMPK. As expected, expression of phosphorylation of S6K1 was partially recovered in Rh30 cells expressing HA-tagged dominant negative AMPKα (Ad-AMPKα-dn), but not GFP (Ad-GFP), and the inhibition of 4E-BP1 was also reversed (Fig. 6a). Further results also indicated that compared with lentiviral sh-GFP, a significant reversed effect on phosphorylation of S6K1 and 4E-BP1 was detected in lentiviral sh-AMPKα Rh30 cells treated by 5 μmol/L and 10 μmol/L CPT (Fig. 6b). Both above-mentioned results implied that CPT inhibits mTORC1 dependent on AMPK.

### AMPK-knockout cells confer resistance to CPT

**Fig. 7:**
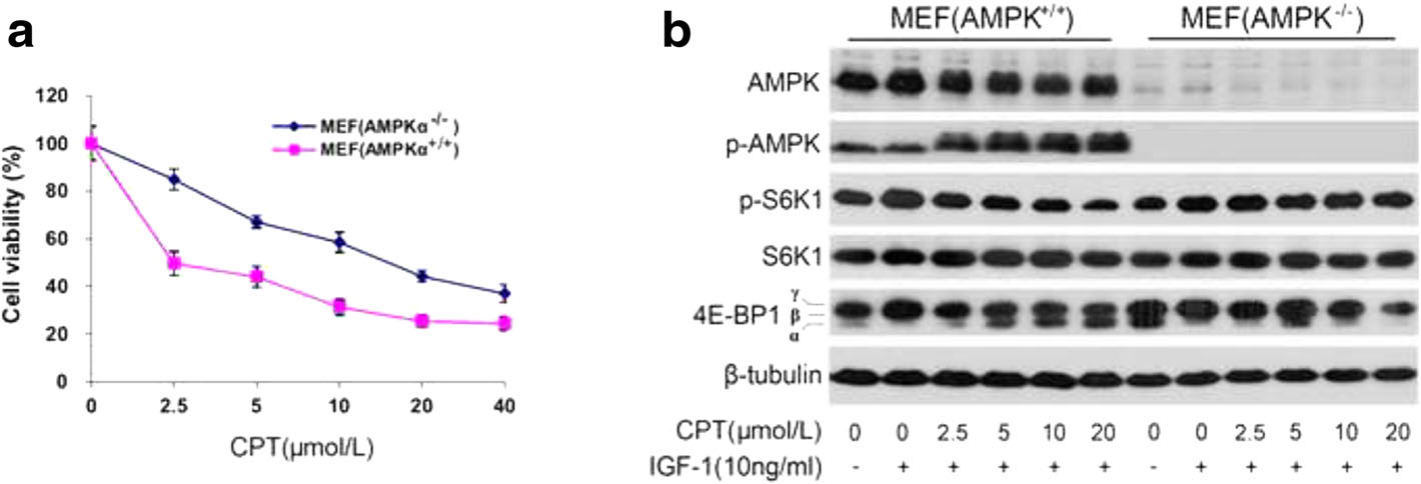
Depletion of AMPKα renders resistance to CPT inhibition of mTORC1 signaling. **a** AMPKα positive MEF cells and AMPKα negative MEF cells were seeded in 96-well plates (10^4^ cells/well) respectively and grown overnight. After 48 h treatment by CPT, each well was added 20 μL of one solution reagent and incubated for 4 h. Cell proliferation was determined by measuring the optical density (OD) at 490 nm using a Wallac 1420 Multilabel Counter**;** (**b**) Serum-starved AMPKα knocked out MEF cells, were firstly exposed to CPT (0-20 μmol/L) for 2 h, then stimulated with IGF-1 (10 ng/mL) for 1 h, followed by western blotting assay for proteins expression of AMPK, p-AMPK, p-S6K1, 4E-BP1 and β-tubulin. MEF cells expressing AMPK as a control

Herein, compared with AMPKα positive MEF cells, AMPKα negative MEF cells shows more resistant to CPT with IC_50_ from 2.45 μmol/L (AMPKα^+/+^) up to 16.14 μmol/L (AMPKα^-/-^) (Fig. 7a). In addition, phosphorylation of S6K1 and 4E-BP1 expressed obviously different between two types of cells. In absence of AMPK mediation, CPT shows no inhibitory effect on expression of p-S6K1 and can’t induce 4E-BP1 band shift, which is contrast to the presence of AMPK (Fig. 7b). This confirmative data further suggested that AMPK mediates the inhibition of CPT on mTORC1 signaling pathway.

### Activation of TSC2 involved in CPT inhibition on mTORC1

**Fig. 8:**
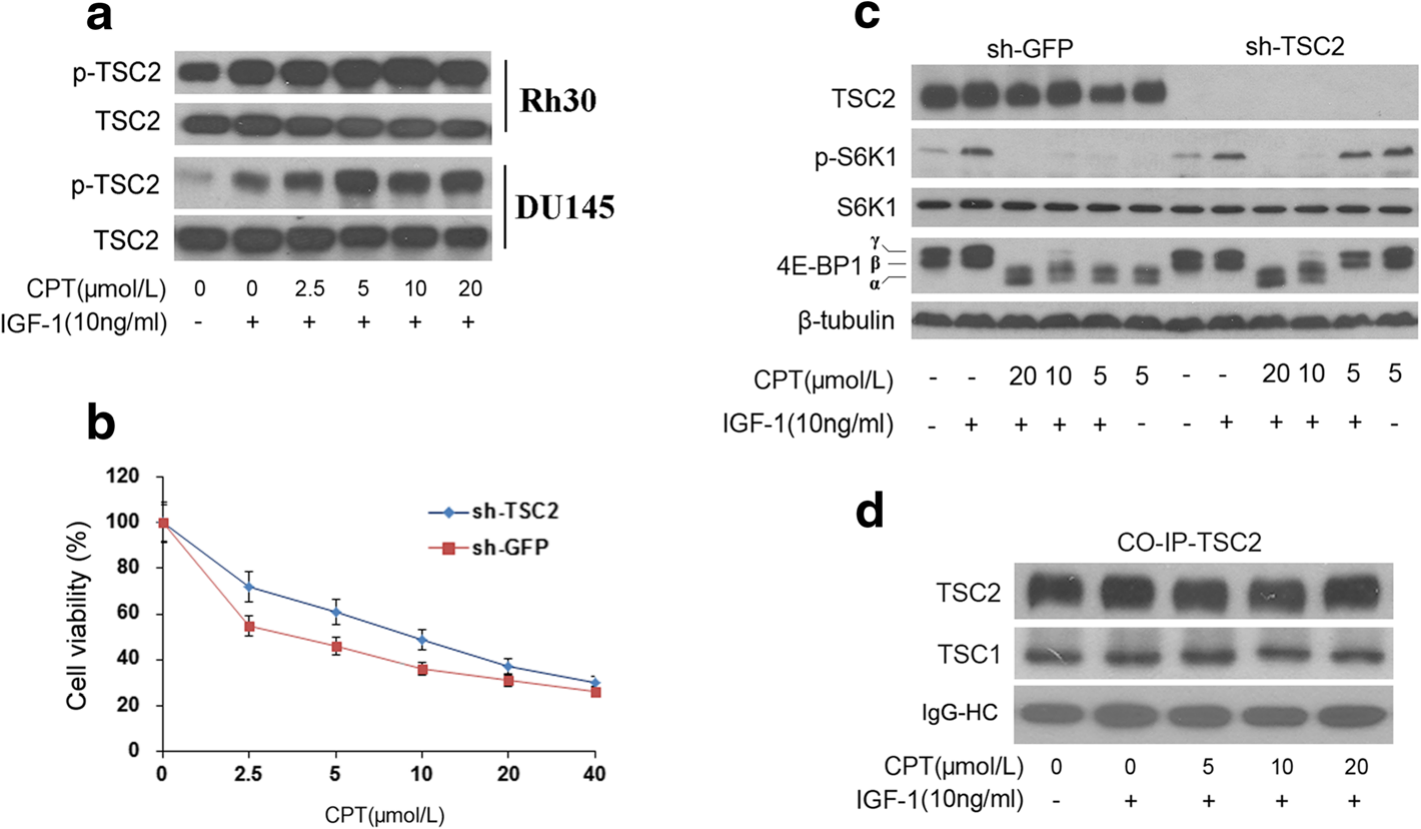
Activation of TSC2 involved in CPT inhibition of mTORC1 signaling. **a** Serum-starved Rh30 and DU145 cells were firstly exposed to CPT (0-20 μmol/L) for 2 h, then stimulated with (10 ng/mL) for 1 h, followed by western blotting assay for proteins expression. Rh30 cells respectively transfected with sh-TSC2 or sh-GFP were: (**b**) seeded in 96-well plates (10^4^ cells/well) respectively and then followed by cell proliferation assay; (**c**) starved for serum and pretreated with CPT (0-20 μmol/L) for 2 h, and then stimulated with (10 ng/mL) for 1 h, followed by western blotting assay for proteins expression. **d** Co-immunoprecipitation assay was executed for TSC2 and TSC1. Note: IgG-HC, heavy chain of IgG

TSC2 as a downstream of AMPK, was associated with TSC1 and formed a functional complex that inhibits mTOR [21]. And the inhibition is mediated by Rheb (Ras Homolog Enriched in Brain), a Ras family small GTPase. The Rheb is inactivated by TSC2 with GAP activity responsible for hydrolysis of RHEB, resulting in mTOR signaling inhibition [21, 22]. Thus, TSC2 is directly phosphorylated by activation of AMPK and its GAP activity is thereby enhanced, leading to hydrolysis of RHEB and the inhibition of mTORC1 [22]. Here, we found that CPT activates AMPK with synchronous activation of TSC2 because phosphorylation of TSC2 increases by a concentration-dependent way in Rh30 cells by the treatment of CPT (Fig. 8a). And compared with transfection of sh-GFP, Rh30 cells with TSC2 silence shows a bit of resistance to CPT evidenced by that sh-TSC2 transfected Rh30 cells are less sensitive to 2.5 μmol/L-10 μmol/L CPT (Fig. 8b) and its expression of phosphorylation of S6K1 and 4E-BP1 is significantly higher than sh-GFP transfected cells at 5 μmol/L CPT (Fig. 8c). Furthermore, CPT didn’t affect the expression of TSC1 when immunoprecipitates TSC2 in Rh30 cells (Fig. 8d), verifying no occurrence of disruption of TSC1/TSC2 complex.

## Discussion

It is well-known that mTOR has been extensively considered a very important target for cancer therapy, and many synthesized mTORC1 inhibitors, rapamycin derivatives such as RAD001, CCI-779, have been tested for cancer patients in clinic [1, 23]. Furthermore, besides the mTOR inhibitors, its upstream proteins regulating mTOR are also prevalent in cancer therapy [24]. mTOR receives signaling from multiple pathways including Akt-dependent and AKT-independent mechanisms, in which mitogenic signals, energy, nutrients and so on generally functions as an initiators to activate the mTOR signaling [7]. Of course, devotion to find novel pathways that regulate mTOR could be beneficial to predict new therapeutic targets for cancer.

Recently, many natural compounds from herbal drugs including cryptotanshinone [14], curcumin [25], epigallocatechin gallate [26], resveratrol [27, 28], apigenin [29], quercetin [30, 31] and so on, also have been found to inhibit mTOR pathway and considered a potential mTOR inhibitor. However, all of these compounds are not derivatives of rapamycin and have little similarity with rapamycin in structure. Thus, it is of great importance to elucidate how CPT inhibits mTORC1 signaling, as this will promote to develop more potent new CPT analogues or accelerate the approval of CPT applying in clinic for cancer therapy.

**Fig. 9:**
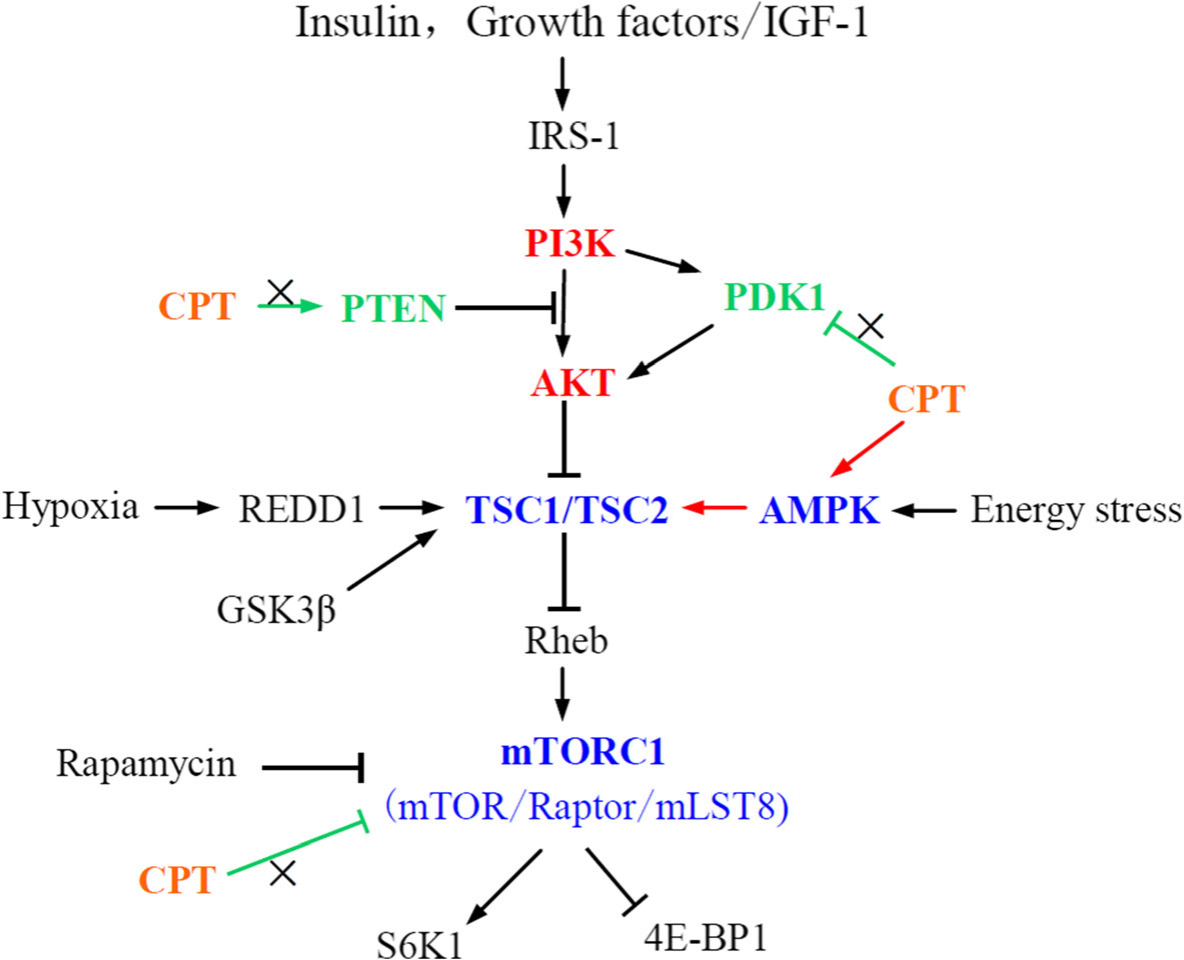
A schematic diagram about CPT inhibition on mTORC1

In this study, it is firstly confirmed that CPT inhibits mTORC1 independent on binding to FKBP12, suggesting the effect of mTOR inhibition was due to signal transduction by regulation of its upstream (Fig. 9). IGF-1 induced AKT-dependent mTOR signal pathway includes PI3K, AKT, PDK-1 and PTEN would finally activate mTORC1 through a series of signal transduction [7]. However, measurement of PI3K indicated that CPT shows no effect on expression of PI3K and its phosphorylation, while phosphorylation of AKT in T308 and S473 residues was activated together [14]. The results implied that growth factors-induced AKT-dependent pathway could not be triggered by CPT, but AKT-independent mTOR signal pathway maybe the target of CPT (Fig. 9).

The main upstream of AKT-independent regulation of mTOR is AMPK/TSC2 pathway [32]. AMPK belongs to a conservative heterotrimeric enzyme complex containing a catalytic subunit and several regulatory subunits, and has been widely considered as an intracellular energy sensor. At the presence of the sorts of stress, energy preservation is promoted by AMPK activation for cell survival responding to energy requirement [11]. Nevertheless, AMPK/mTOR pathway has been concerned in cancer by a lot of investigators because it controls many oncogenic protein transcription and translation [33, 34], highlighting that AMPK, an important energy regulator, was an emerging target for inhibition of the mTOR pathway in cancer [34]. Here, CPT could activate AMPK when inhibiting Rh30 cells proliferation, and pretreatment of compound C or dominant-negative AMPK in Rh30 cells could significantly attenuates the CPT’s inhibitory effect. Additionally, MEF cells with AMPK knocked out shows more resistant to CPT than AMPK positive cells. All of above data entirely demonstrate that CPT is an AMPK activator (Fig. 9). Nowadays, many AMPK activators have been developed to enter the preclinical stage, even clinical stage for cancer including the best described metformin, AICAR, and A-769662 [35]. Metformin, the most widely used anti-diabetic first-line drug all over the world, has reattracted people’s concern and been justified to be a potential anticancer drug by the increasing evidences of anticancer efficacy [36, 37]. Actually, CPT has previously been proved to exert anti-diabetes and anti-obesity effect in mice via activation of AMPK [38]. CPT like metformin shows not only antidiabetic but also anticancer action.

AMPK communicates the inhibitory signal to the mTORC1 by indirect and direct mode. TSC2 is phosphorylated and activated by AMPK mediating indirect inhibition on mTORC1 [39]. Activated TSC2 and TSC1 bind to form a heterodimeric complex to suppress the Rheb, a Ras-related GTPase selectively activating mTORC1 [21]. Moreover, AMPK phosphorylating TSC2 at S1345 could also induce S1341 and S1337 of TSC2 to be phosphorylated by GSK3β, a component of the destruction complex of the canonical Wnt signaling pathway [40]. AMPK and GSK3β simultaneously phosphorylating TSC2 significantly promotes TSC2 activation and inhibition of mTORC1. Actually, TSC2 is phosphorylated by Akt, ERK, AMPK and GSK3β on distinct sites, highlighting the significance of TSC2 in integrating cellular energy signal and growth molecules to regulate protein synthesis [40].

Although TSC2 plays an indispensable role in AMPK inhibition of mTOR, there is an additional mechanism independent of TSC2 between AMPK and the mTOR [20]. It has been demonstrated that AMPK directly phosphorylating raptor should lead to inhibition of mTORC1, and the inhibition was reversed by the AMPK activators AICAR and phenformin [20]. Thus, the TSC2-dependent and -independent way to inhibit mTORC1 by AMPK suggested the possible complexity for CPT inhibition of mTORC1.

In this study, CPT could activate TSC2 when activates AMPK, but has no activation of raptor, clarifying that a TSC2-dependent mechanism was triggered by CPT in cancer cells. On the other hand, previously we also found that CPT could generate plenty of ROS in cancer cells, then induce MAPK cascade pathways including activation of MAPK/P38, MAPK/JNK and inhibition of MAPK/ERK [41], resulting in caspase-independent apoptosis in cancer cells. However, the relationship between ROS and AMPK is mutually dependent. Hypoxia could trigger AMPK activation via generating ROS [42, 43]. Therefore, CPT induction of ROS and subsequent activation of AMPK seems to simulate a course of hypoxia. Taken together, CPT activates AMPK-TSC2 axis to suppress the mTORC1 possibly through inducing a disorder of energy metabolism in cancer cells.

The mTOR enhances tumorigenesis and has become an attractive therapeutic target in cancer. Both rapamycin, a specific inhibitor of mTORC1, and rapamycin derivatives shows highly effective in treating all types of cancer [44]. However, insufficient efficacy and obvious side effects in clinical trials may limit the development and application of these drugs as anticancer agents [45, 46]. Therefore, targeting upstream signaling pathways that control mTOR may facilitate to find more sensitive targets for inhibiting cancer [33, 34, 47]. Thus, we believed that natural compounds specifically targeting upstream of mTOR should be promising anticancer agents. Here, CPT highly activates AMPK in cancer cells and significantly inhibits mTORC1 through AMPK-TSC2 axis, indicating that CPT is a natural potent AMPK activator and suitable for clinical cancer therapy.

## Conclusion

In this study, we found that CPT inhibition of mTORC1 is via activating AMPK-TSC2 axis rather than other mTORC1-related molecules. It suggested that CPT is a potent AMPK activator and should become a potential anticancer agent in clinic.

## Abbreviations

4E-BP1: Eukaryotic initiation factor 4E binding protein 1
AMPK: Adenosine monophosphate-activated protein kinase
CPT: Cryptotanshinone
mTORC1: The mammalian target of rapamycin complex 1
PIKK: Phosphatidylinositol 3-kinase-related kinase
ROS: Reactive oxygen species
S6K1: p70 S6 kinase 1
TSC: Tuberous sclerosis complex
