# Correction to: Cryptotanshinone activates AMPK-TSC2 axis leading to inhibition of mTORC1 signaling in cancer cells

**DOI:** 10.1186/s12885-019-5458-y

**Published:** 2019-03-21

**Authors:** Wenxing Chen, Yanhong Pan, Siliang Wang, Yuping Liu, Guangying Chen, Liang Zhou, Chao Zhang, Wenting Ni, Aiyun Wang, Yin Lu, Shile Huang

**Affiliations:** 1 School of Pharmacy, Nanjing University of Chinese Medicine, NO.138, Xianlin Avenue, Nanjing, 210023 Jiangsu Province China; 2 Jiangsu Key Laboratory for Pharmacology and Safety Evaluation of Chinese Materia Medica, Nanjing University of Chinese Medicine, Nanjing, 210023 Jiangsu Province China; 3 College of Chemistry and Chemical Engineering, Hainan Normal University, Haikou, 571158 China; 4 Louisiana State University Health Sciences Center, Shreveport, LA USA

**Correction to: BMC Cancer (2017) 17:34**

**https://doi.org/10.1186/s12885-016-3038-y**

Following publication of the original article [1], the author noticed the following errors in the article.

1) The following authors were mistakenly omitted from the published article:

- Dr. Shile Huang as lead corresponding author
- Dr. Chao Zhang as co-author

The correct authorship is as follows:

Wenxing Chen^1,2^, Yanhong Pan^1^, Siliang Wang^1^, Yuping Liu^1^, Guangying Chen^3^, Liang Zhou^1^, **Chao Zhang**^**4**^, Wenting Ni^1^, Aiyun Wang^1^, **Yin Lu**^1,2^* and **Shile Huang**^4^*.

* Correspondence: shuan1@lsuhsc.edu and luyingreen@126.com

2) Correction to Acknowledgement statement. The correct statement is as follows:

This work was supported in part by Natural Science Foundation of Jiangsu Province (BK2012854, W. Chen), National Natural Science Foundation of China (81673648, W. Chen;81173174, Y. Lu; 21162009, G. Chen), the National Institutes of Health NCI CA115414, S. Huang), American Cancer Society (RSG-08-135-01-CNE, S. Huang), Feist- Weiller Cancer Center, Louisiana State University Health Sciences Center, and a project of the Priority Academic Program Development of Jiangsu Higher Education Institutions (PAPD), Qing Lan Project of Jiangsu and a preliminary foundation of NJUCM (13XYYZ4).

3) Correction to Authors’ contribution statement. The correct statement is as follows:

WXC, YL, and SH conceived the project and designed the research. WXC performed adenoviral and lentiviral assays, and YHP, YPL, WTN, and CZ conducted cell culture and western blot. SLW conducted the molecular docking. LZ prepared the figures and performed the statistical analysis. AYW, GYC, and YL participated in coordination. WXC wrote the manuscript. All authors read and approved the final manuscript.

4) Figure correction: The authors noticed an error in Fig. 8a. Please see the correct Fig. 8a and figure legend below.

**Fig. 8:**
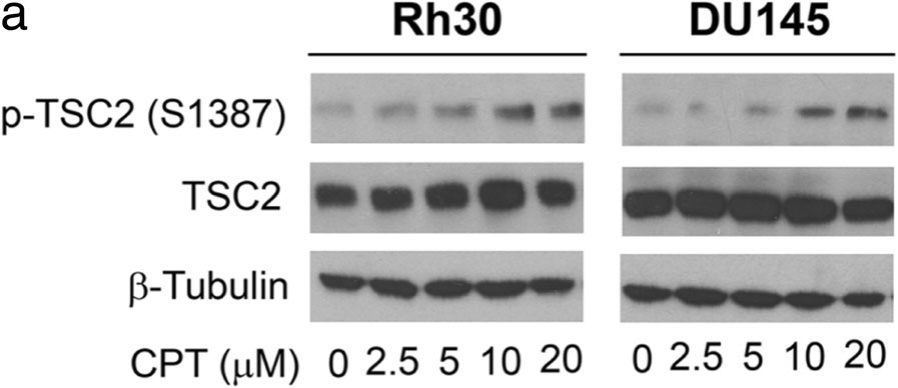
Activation of TSC2 involved in CPT inhibition of mTORC1 signaling. **a** Rh30 and DU145 cells were exposed to CPT (0–20 μmol/L) for 24 h, followed by western blotting with indicated antibodies

The authors apologize for any inconvenience caused.
